# A Calibration-Free pH Sensor Using an In-Situ Modified Ir Electrode for Bespoke Application in Seawater

**DOI:** 10.3390/s22093286

**Published:** 2022-04-25

**Authors:** Yuqi Chen, Richard Compton

**Affiliations:** Physical & Theoretical Chemistry Laboratory, University of Oxford, Oxford OX1 3QZ, UK; yuqi.chen@sjc.ox.ac.uk

**Keywords:** calibration-free, pH sensor, in-situ modification, Ir electrode, seawater

## Abstract

A bespoke calibration-free pH sensor using an in situ modified Ir electrode for applications in seawater is reported. The electrochemical behaviour of an iridium wire in air-saturated synthetic seawater was studied and the formation of pH-sensitive surface layers was observed that featured three pH-sensitive redox couples, Ir(III/IV), ${\mathrm{IrO}}_{x}O^{I-}/{\mathrm{IrO}}_{x}O^{\mathrm{II}-}H,$ and H_upd_/H^+^, where H_upd_ is adsorbed hydrogen deposited at underpotential conditions. The amperometric properties of the electrochemically activated Ir wire were investigated using linear sweep voltammetry first, followed, second, by square wave voltammetry with the formation conditions in seawater for the optimal pH sensitivity of the redox couples identified. The sensor was designed to be calibration-free by measuring the “super-Nernstian” response, in excess of ca 60 mV per pH unit, of Ir(III/IV) relative to the less sensitive upd H oxidation signal with the pH reported on the total pH scale. The pH dependency of the optimised sensor was 70.1 ± 1.4 mV per pH unit at 25 °C, showing a super-Nernstian response of high sensitivity.

## 1. Introduction

pH measurements in chemistry are ubiquitous. As defined by IUPAC [1], pH is a function of the activity of the hydrogen ion in a solution:

(1) $$\mathrm{pH}=-\log a_{H^{+}}=-\log\left(\frac{m_{H^{+}}\gamma_{H^{+}}}{m^{\theta }}\right)$$

where $a_{H^{+}}$ is the single ion activity (measured on the molality scale in mol kg^−^^1^), $\gamma_{H^{+}}$ is the activity coefficient of the hydrogen ion (H^+^) at a molality of $m_{H^{+}}$, and $m^{\theta }$ is the standard molality (1 mol kg^−^^1^). Note that the pH is defined by IUPAC in terms of the single ion quantities and so IUPAC regard Equation (1) as a ‘notional definition’. The development of primary pH standards utilises a ‘primary method of measurement’ that is based on the Harned cell, which comprises a hydrogen electrode and a silver/silver-chloride electrode in a cell containing hydrochloric acid electrolyte and without a liquid junction [2]. Debye–Hückel theory [3] is used for the required chloride ion activity coefficient estimation via the Bates–Guggenheim convention with the latter restricted to ionic strengths of less than 0.1 mol kg^−^^1^ [4]. This leads to uncertainties of at least 0.003 in pH. It is noteworthy that the seawater has a high ionic strength (I ~0.7 mol kg^−^^1^) [5] so that pH measurements in seawater are expected to require specific procedures.

Seawater comprises about 97.2 percent of the Earth’s known water and covers approximately 71 percent of its surface. Seawater compositions vary with their biological content, as well as reflecting local coastal industry and geology [6]. pH is an important oceanographic parameter, which is essential for investigating the dynamic state of the chemical and biological processes. First, there is a close interplay between pH, chlorophyll-a (chl-a), and dissolved oxygen (DO) [7]. The pH value of seawater is normally 8.1 but can be higher for eutrophic waters [8] because of the emission of nitrogen- and phosphorous-containing species resulting from human activities [9]. A high pH may inhibit the photosynthesis of algae [10,11]. Chl-a is an important indicator for the presence of algae, notably for assessing eutrophication, and, via satellite imaging of fluorescence from plankton, concentrations. pH variations can thus reflect direct changes in chl-a concentration [7]. Note that phytoplankton are made up of both single-celled algae and cyanobacteria [12], the concentration of which can be monitored at the single entity level by fluoro-electrochemical microscopy [13]. Second, and of current vital importance, the pH of seawater is a significant reference for ocean acidification as a result of carbon emission and reflects its ecological effects [9,14]. Carbon uptake via seawater is a major sink of ${\mathrm{CO}}_{2}$, during which the formation of carbonic acid from atmospheric carbon dioxide lowers the pH of seawater (Equation (2)). For example, the pH of seawater is thought to have decreased from 8.2 before the Industrial Revolution in Western Europe to about 8.1 today [9].

(2) $${\mathrm{CO}}_{2}+H_{2}O\to H_{2}{\mathrm{CO}}_{3}$$

Meanwhile, carbonate in seawater is important for marine life to build shells and skeletons. Under conditions of severe acidification, shells and skeletons can dissolve (Equation (3)).

(3) $$H_{2}{\mathrm{CO}}_{3}+{\mathrm{CO}}_{3}^{2-}\to \;2{\mathrm{HCO}}_{3}^{-}$$

Third, pH is also directly linked to the solubility of heavy metals [15,16]. The lower the pH, the more toxic the water possibly is as the metals tend to be more soluble. Thus, significant attention needs to be paid to the pH variation of seawater in order to maintain the environmental ecological balance.

The concept and measurement of pH was initiated over a century ago [17] by Sorensen working in the Carlsberg Laboratory, leading ultimately to the IUPAC definition. The latter, however, is not recommended for seawater because of its high ionic strength. Instead, three scales were developed using proton concentration scales rather than the activity scale as defined by IUPAC [18,19,20,21,22]. Specifically, the three scales are the free hydrogen ion scale (Equation (4)), the total hydrogen ion scale (Equation (5)) and the seawater scale.

The free hydrogen ion scale [23,24,25] is defined by:

(4) $${\mathrm{pH}}_{F}=-\log m_{H^{+}}$$

where the total amount of H^+^ is calculated in terms of its concentration (molality) rather than activity. In contrast, the total hydrogen ion scale accounts better for the complex chemical environment of seawater in which ${\mathrm{SO}}_{4}^{2-}$ ions, if present, can react with H^+^ to form the ion ${\mathrm{HSO}}_{4}^{-}$ [19,26], so in terms of the addition of HCl, the resulting H^+^ concentration is less since some protons form ${\mathrm{HSO}}_{4}^{-}$. With this definition:

(5) $${\mathrm{pH}}_{T}=-\log m_{H^{+}}-\log m_{\mathrm{HSO}4^{-}}=-\log\left\{m_{H^{+}}\left[1+m\left({\mathrm{SO}}_{4}^{2-}\right)/K(m_{{\mathrm{HSO}}_{4}^{-}}\right)]\right\}$$

where $m_{{\mathrm{SO}}_{4}^{2-}}$ is the stoichiometric concentration of sulphate and K(${\mathrm{HSO}}_{4}^{-}$) is the dissociation constant for bisulphate ion [18,21,25,27,28].

The seawater scale recognises the possible presence of both sulphate and fluoride ions [27,29], but this scale was suggested to be unhelpful by Dickson [30] in 1993, who suggested that fluoride should simply be treated as a minor acid base species [18]. Clearly, however, in reporting seawater measurement data it is necessary to state which units and scales are used.

Millero et al. [18] proposed an experimental approach in which pH was studied in terms of the proton concentration with a unit of kg-H_2_O^−1^, while the buffers were prepared in seawater using Bis(2-amino-2-methyl-1,3-propaneldiol), Tris(2-amino-2-hydroxymethyl-1,3-propaneldiol), Morpholine, or 2-Aminopyridine referring to the recipes suggested by Bates and co-workers [23,31,32,33]. Then, the corresponding potentials of the buffers with different pH were measured with a Harned cell approach and the resultant pH values were in good agreement with the total pH scale [30,31] Bates’s work provided a good insight for the results discussed below; Tris/HCl buffers prepared in synthetic seawater are applied for the experiments reported later in this paper.

In addition to optical fibre sensing applied for marine environment monitoring [34], spectrophotometry is commonly used to measure the pH of seawater [35,36]. Spectrophotometry is based on different absorbance characteristics of the basic and acidic forms of sulfonephthalein indicators, L^2−^ and HL^−^ (from the secondary dissociation ${\mathrm{HL}}^{-}\overset{K_{2}}{↔}H^{+}+L^{2-}$), the relative amounts of which alter measurably within the range of pH values seen in normal seawater environments. The selection of the specific indicator used, commonly a sulfonephthalein derivative, ref. [37] is decided by the specific pH range of candidate analyte. The log of the secondary dissociation constant (log K_2_) of the sulfonephthalein indicator should be comparable to the expected pH of the sample solution, i.e., (log K_2_ (indicator)−1) < pH (sample) ≤ log K_2_ (indicator) [36,38]. For example, bromocresol green (K_2_ ≈ 10^−4.4^) [38,39] is appropriate for acidified samples used in determinations of seawater alkalinity [40], while thymol blue (K_1_ ≈ 10^−8.6^) [41] is most appropriate for surface waters, where generally 7.90 ≤ pH ≤ 8.40 [36,42]. The relationship between the pH and the measured parameters is represented by Equation (6):

(6) $$\mathrm{pH}={\mathrm{pH}}_{2}+\log\left[L^{2-}\right]/\left[{\mathrm{HL}}^{-}\right]={\mathrm{pH}}_{2}+\log\left(\frac{A_{\lambda }-A_{\lambda_{\mathrm{Min}}}}{A_{\lambda_{\mathrm{Max}}}-A_{\lambda }}\right)$$

where ${\mathrm{pH}}_{2}=-\log K_{2}$, $K_{2}$ is the HL^−^ dissociation constant; $A_{\lambda }$ is the absorbance at wavelength λ and is related to pathlength (l), total indicator concentration (D_T_) and molar absorbance ($a_{\lambda }$) through the well-known Beer-Lambert relationship $A_{\lambda }=a_{\lambda }{*D}_{T}*l$ [38,41].

It is noteworthy that spectrophotometry can realise measurements to within ±0.001 pH units while potentiometry has a precision no better than ±0.02 pH units depending on multiple parameters [20,43]. In terms of potentiometric techniques for pH sensing, a H^+^ ion-selective glass electrode-based pH meter is common in the laboratory. However, it requires regular calibrations by standard buffers. More importantly, the use of low ionic strength buffers to calibrate a glass electrode for use in high ionic strength solutions, namely seawater, may cause errors [19,21]. Beyond the glass pH meter, in the case of potentiometric titrations, an all-solid-state ion-selective electrode (ISE), with functionalised multiwalled carbon nanotubes being drop casted on a glassy carbon electrode, were developed by Cuartero et al. [44]. This ISE was applied in a 600 mM NaCl to mimic seawater environments. However, similarly to the glass pH meter, calibrations are recommended to be made every hour prior to and during the measurements to compensate for electrode drift and for changes in temperature. Finally, pH is calculated referring to the measured potential E using the calibrated linear relationship (Equation (7)):

(7) $$E=E^{0}+s\times \log\left[a_{1}\left(H^{+}\right)\right]$$

where the slope s is equal to 2.303 $\times \frac{\mathrm{RT}}{\mathrm{zF}}$ (R is the gas constant, T is the temperature, z is the charge of the ion, and F the Faraday constant) based on the Nernst equation [44].

**Figure 1:**
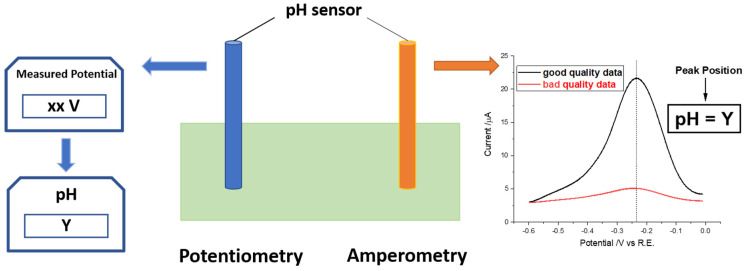
A schematic diagram to contrast potentiometric and amperometric measurements (see text).

Even when conducted with the greatest expertise and diligence, potentiometry simply reports a single number from which it is often difficult, if not impossible, to ascertain the quality or validity of the measurement (Figure 1). This consideration is especially important in complex matrixes such as seawater and blood where electrode fouling is often encountered. In response to this need, we have suggested the use of voltammetry where the response in the form of a current-voltage plot allows for a measure of the quality of response to be judged [45,46,47,48]. The concept is shown schematically in Figure 1 from which it is apparent that the peak shape and width allow for a measure of measurement ’quality’ and for deciding whether the electrode needs to be repositioned, cleaned, or replaced. For example, as shown by the right part of Figure 1, the black voltammogram obtained by amperometry is better-defined than the red one and the associated measurement is more reliable.

Whilst amperometric pH sensors have found wide application [49,50,51,52,53,54,55], there has been only limited application of amperometric pH measurements in seawater, although Sisodia et al. [56] recently reported an electropolymerised 2-(methylthio)phenol modified glassy carbon based electrode as an voltammetric pH sensor in seawater that had a sub-Nernstian response in buffers (pH = 4–9.2) of 51 mV/pH unit. The measured pH (8.28) of seawater using the electrode had a good match compared to that obtained by a conventional glass pH probe (8.30).

In this paper we develop a metal oxide microelectrode for amperometric pH detection based on pH-sensitive anodic iridium oxide film (AIROF) synthesised by cyclic voltammetry in seawater on the surface of an iridium wire. Noteworthy is that in contrast to the iridium oxide with a near-Nernstian response (ca. 60 mV per pH unit) prepared by other methods, e.g., sol-gel [57,58] chemistry, sputtering [59,60], and thermal methods [61,62], AIROF formed on the bespoke electrode is able to respond with a super-Nernstian slope as reported [63,64,65]. The observed pH responses are summarised in Table S1 Supplementary Materials. Second, the bespoke sensor is calibration-free. All the electrochemical reactions investigated in this project take place in a three-electrode system [66]. The basis of calibration free amperometric pH measurement is the recording of two or more voltammetric peaks with different sensitivity to pH. Then the *difference* of the associated peak potentials, if measured in the same voltammogram at essentially the same time, is independent of any drift for example in the reference potential [49,52,67,68]. In the case of the iridium wire-based sensors, the analytical pH responses arise from the pH dependency of the potential of the strongly pH sensitive Ir^3+/4+^ redox couple measured relative to the much less pH-sensitive hydrogen underpotential re-oxidation. As the potentials of these redox reactions are collected during one measurement and only the *relative* potentials between them are used analytically, the stability and accuracy of the reference electrode are not important. Linear sweep voltammetry was investigated first, followed by square wave voltammetry to explore the relative sensitivity of the pH dependency to the two techniques. The super-Nernstian relationship coupled with the capability of assessing the measurement quality imply the validity and merit of the bespoke sensor.

## 2. Experimental Section

Chemicals and Reagents. Solutions were prepared using deionised water with a resistivity of 18.2 MΩ cm at 298 K (Millipore, Millipak Express 20, Watford, UK). All chemicals were of analytical grade and were used as received without any further purification. Three synthetic seawater samples with defined pH values were prepared for the calibration of a HACH LANGE Sension^+^ PH31 pH meter, one of 2-Aminopyridine (99.0%, Sigma-Aldrich, Saint Louis, MO, USA), Tris(hydroxymethyl) Aminomethane (Tris, 99.0%, Sigma-Aldrich, Saint Louis, MO, USA), and 2-Amino-2-methyl-1,3-propanediol (Bis, >99%, Alfa Aesar, Heysham, Lancashire, UK) was dissolved in synthetic seawater separately with a molarity of 0.08 M. These solutions have been shown to give good correlation with the total pH scale as discussed above [18,30]. The composition of synthetic seawater and corresponding buffers is presented in Table 1 following a literature recipe [30]. Sodium chloride (NaCl, 99.5%), potassium chloride (KCl, 99.5%), magnesium chloride (MgCl_2_, 98%), calcium chloride (CaCl_2_, 97%), and sodium sulphate (Na_2_SO_4_, 99%) were purchased from Sigma-Aldrich. The 0.04 M equimolar buffers were finally obtained by adding 0.04 M hydrochloric acid (Fisher Scientific UK Limited, Loughborough, Leicestershire, UK~37%) to synthetic seawater. The pH values of ‘standard seawater buffers’ were defined as 6.77, 8.07, and 8.81, respectively, for 2-Aminopyridine, Tris, and Bis. To study the pH dependency of the bespoke electrode, various buffer solutions were prepared with their pH values adjusted by adding a trace of HCl.

Electrochemical apparatus and methods. Electrochemical measurements were performed using a μAutolab II potentiostat (Metrohm-Autolab BV, Utrecht, The Netherlands). A standard three-electrode set-up was used, consisting of a saturated calomel reference electrode (SCE + 0.244 V vs. SHE, BASi Inc., West Lafayette, IN, USA), a graphite rod counter electrode, and an iridium wire (0.1 mm in diameter, GoodFellow, UK) as the working electrode. The Ir electrode was pretreated by heating the metal using a Bunsen burner for 10 s to remove surface contamination and impurities. The electrochemical set up was thermostated at a constant value of 25.0 ± 0.2 °C. High purity N_2_ flow (BOC Gases plc, UK) was used to remove oxygen from aqueous solutions as needed prior to the electrochemical measurements. Cyclic voltammetry (CV) was used to study the electrochemical behaviour of the Ir electrode and for the potential cycling activation. Linear sweep voltammetry (LSV) and square wave voltammetry (SWV) were conducted to determine the pH dependency of in situ modified Ir wire after a potential cycling activation.

## 3. Results and Discussion

In the following sections, we first analyse the voltammetry of an iridium wire in synthetic seawater under conditions of controlled pH. We demonstrate that it is possible to reproducibly form layers of iridium oxide in synthetic seawater and assign the various pH-sensitive redox couples, which are subsequently used as the basis for the amperometric calibration-free pH sensing without the need for any degassing to remove dissolved oxygen. Next, potential cycling is developed as a simple method of electrode activation directly within seawater and this is optimised in terms of the potential window used. Further electrode optimisation is made in respect of recording the various relevant redox couples pertinent to pH measurements and characterising the corresponding pH dependency of a calibration-free sensor. Linear sweep voltammetry was investigated first, followed by square wave voltammetry to improve sensitivity and precision.

### 3.1. Cyclic Voltammetry of Iridium and Iridium Oxides

**Figure 2:**
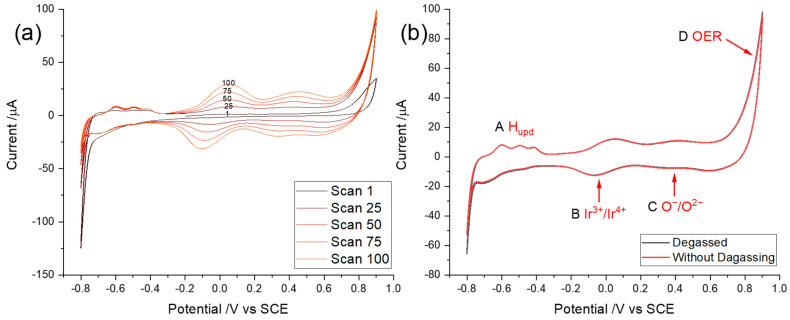
(**a**) Cyclic voltammograms showing the activation of an Ir wire at a scan rate of 0.5 Vs^−1^ for multiple cycles. The start potential was −0.2 V vs. SCE in air-saturated synthetic seawater with pH = 8.1. (**b**) Overlaid 40th cyclic voltammograms in synthetic seawater with pH = 8.1 using an Ir wire at a scan rate of 0.5 Vs^−1^; degassed: black line, without degassing: red line.

Cyclic voltammetry was conducted to study the formation of iridium oxides on the surface of an iridium wire and other redox reactions that occur during potential cycling. It was discovered that to ensure reproducible data, the surface of the Ir wire should be free of oxide prior to voltammetric measurements. Accordingly, as reported in previously [48], the Ir surface was treated by pre-flaming to renew the surface of the metal electrode between experiments. To explore the voltammetric behaviour of an Ir wire and the effect of degassing, the pre-flamed Ir electrode was first immersed in air-saturated synthetic seawater buffered by Tris(hydroxymethyl) Aminomethane/HCl to give a pH close to typical natural seawater (8.1 [9]), and then the same process was repeated in a degassed solution. Cyclic voltammetry was conducted for 100 cycles starting at a potential of −0.2 V vs. SCE with scan reversal at a potential of 0.9 V with a subsequent sweep to −0.8 V vs. SCE where the potential was again reversed as shown in Figure 2a. In Figure 2b, the 40th scan of cyclic voltammograms obtained in synthetic seawater with and without degassing are overlaid. It is thought that this procedure leads to the steady build-up of a surface layer, the thickness of which increases with each potential cycle, which displays several redox features in conventional electrolytes [69,70,71] and are closely mirrored in the data obtained in seawater as shown in Figure 2. Note that comparison of the data with and without degassing shows no difference of peak shape or numbers of peaks between the two, with four clearly discernible voltammetric features labelled as A, B, C, and D. This comparison implies that degassing has no effect on the voltammetry and represents an important step in respect of developing amperometric pH measurements for direct use in seawater without the need for removal of oxygen from dissolved air.

Feature A was assigned to the re-oxidation of underpotential deposited (UPD) adsorbed hydrogen, H_upd_, formed at very negative potentials (more negative than −0.32 V vs. SCE). It is notable that it is a one proton–one electron transfer reaction with the reductive formation described by Equation (8) and the oxidative desorption by Equation (9) [72,73,74,75]:

(8) $$M\left(s\right)+H_{3}O^{+}+e^{-}⇌M-H_{\mathrm{ad}}+H_{2}O⇌\frac{1}{2}H_{2}+M-H_{2}O_{\mathrm{ad}}$$

(9) $$M-H_{\mathrm{ad}}⇌H^{+}+e^{-}+M$$

where M is an empty adsorption site on the surface.

Feature B was attributed to an Ir(III/IV) redox transition, previously confirmed by XPS [48,70,71,76], associated with the formation of hydrous oxides, where the oxidation peak is at a potential of ca. 0.08 V vs. SCE and the reduction peak is at ca −0.04 V vs. SCE. Note that this process involves various possible redox reactions and the exact stoichiometric composition of the hydrous film is reported as hard to determine [47,65,77]. The redox process is known to involve numbers of electrons and protons with a ratio of 2:3 leading to a “super-Nernstian” pH dependency of ca 89 mV per pH unit [47,78,79], as implied in the reaction suggested by Olthuis et al. [65]:

(10) $${\mathrm{Ir}}_{2}O{\left(\mathrm{OH}\right)}_{3}O_{3}{}^{3-}+3H^{+}+2e^{-}⇌2\mathrm{Ir}{\left(\mathrm{OH}\right)}_{2}O^{-}+H_{2}O$$

where “super-Nernstian” means a response of greater than 60 mV per pH unit. The average transfer of 1.5 protons per electron are understood in terms of two iridium ions each gaining an electron and the associated oxide ions gaining three. Note that the redox reaction between ${\mathrm{Ir}}_{2}O{\left(\mathrm{OH}\right)}_{3}O_{3}{}^{3-}$ and $\mathrm{Ir}{\left(\mathrm{OH}\right)}_{2}O^{-}$ is denoted as Ir(III/IV) for simplification in this paper.

Feature C shows an oxidation peak at about 0.42 V with the corresponding reductive peak at 0.37 V vs. SCE. Pickup et al. [80] and Kasian et al. [81] suggested that the redox couple in Feature C can be attributed to further oxidation of the Ir hydrous oxides, e.g., from Ir (IV) to Ir (V/VI), while Pfeifer et al. [70,71,76] assigned it to the oxidation of the oxide anion O^2^^−^, contained in the IrO_x_ matrix in form of adsorbed OH groups, to O^−^:

(11) $${\mathrm{IrO}}_{x}O^{\mathrm{II}-}H⇌{\mathrm{IrO}}_{x}O^{I-}+H^{+}+e^{-}$$

Feature D is related to the oxygen evolution reaction (OER) [70]:

(12) $$2H_{2}O\to O_{2}+4H^{+}+4e^{-}$$

As the number of potential cycles increases as shown in Figure 2a, a build-up of the Ir hydrous oxide layer was inferred with scans increasing because of the repeated redox process as explained by the mechanism reported in the previous paper [48]. It is noteworthy that the four features are all pH sensitive but have different pH dependencies, which is significant for the development below of a bespoke pH sensor for seawater in respect to facilitating calibration-free measurements.

### 3.2. Optimization of Potential Cycling Activation

The different redox processes encountered during potential cycling were identified and explained in the previous section. To obtain better resolved pH-sensitive redox couples and improve the sensitivity in respect of pH detection, the most effective potential window of potential cycling was studied in the following. To be specific, the effect of the cathodic limit potential was investigated first, followed by that of the anodic limit potential.

**Figure 3:**
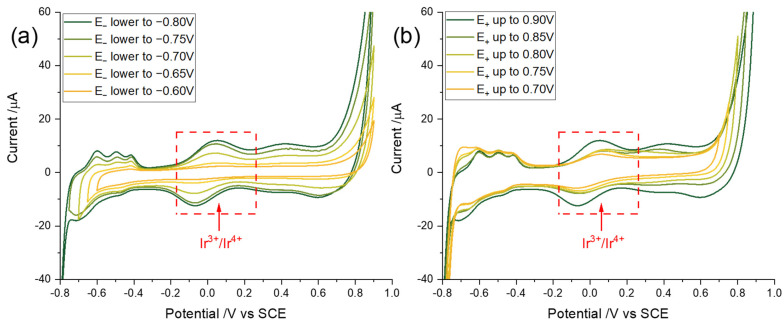
Cyclic voltammograms of the 40th cycle SCE in air-saturated synthetic seawater with pH = 8.14 starting at a potential of −0.2 V for activation of an Ir wire with different (**a**) cathodic and (**b**) anodic potentials at a scan rate of 0.5 Vs^−1^.

As shown in Figure 3a, cyclic voltammograms with different potential windows using a pre-flamed Ir wire were measured at a scan rate of 0.5 Vs^−1^ in an air-saturated Tris/HCl solution prepared in synthetic seawater (pH = 8.14, corresponding to natural seawater [9]). Note that as the potential window shifts as pH changes, conducting the optimisation in synthetic seawater of a typical and average pH results in a potential window applicable to a wide range of seawaters, the pH of which can vary from 7.5 to 8.5 depending on the local conditions [43]. The cyclic voltammetry starts at a potential of −0.2 V and is first swept anodically to a fixed potential of 0.9 V, then swept to different cathodic limits varying from −0.8 V to −0.6 V vs. SCE. To find the optimised cathodic potential, the 40th cycles of each voltammogram were displayed in Figure 3a. Figure 3b shows the 40th scans of the CVs with the cathodic potential being fixed at −0.8 V, while the anodic potential was progressively increased to 0.7 V from 0.9 V vs. SCE.

The signal to the background level of the most dominant Ir(III/IV) redox couple is assigned to be the reference for determining the effectiveness of the potential window as it is the best defined and most prominent. The effectiveness was judged on the basis that the higher the signal to background level, the better for pH detection. In Figure 3a, the redox couple of the 40th cycle becomes better defined with a reductive limit of −0.8 V whilst, in Figure 3b, the highest resolution is achieved with an anodic potential limit extended to 0.9 V. It is significant that the anodic limit has a greater effect on Feature C compared to the cathodic limit. To be more specific, the trend for Feature C to disappear was more obvious as the anodic side was narrowed while the Feature B stayed relatively constant. Pfeifer et al. [70] reported that the IrO_x_O^I−^ formed during the redox reaction in Feature C is the catalyst for OER (Equation (13)):

(13) $$\begin{array}{cc} {\mathrm{IrO}}_{x}O^{I-}+H_{2}O & \to {\mathrm{IrO}}_{x}O^{I-}-O^{I-}-H+H^{+}+e^{-}\\ & \to {\mathrm{IrO}}_{x}+O_{2}+2H^{+}+3e^{-} \end{array}$$

Thus, the redox feature of interest correlates with the onset of the ORR. Meanwhile, the trend of signal to background level improvement gets less significant when the potential limit is extended either more anodically or cathodically, consistent with literature reports [80,82]. Noting that Pickup et al. [80] reported that the hydrous oxide begins to dissolve when more positive potentials were applied, so no further extension of the potential window was explored. Considering the data in Figure 3, we infer that potential sweeps within the ranges (A) −0.6 V to −0.8 V (H_upd_ region) and (B) 0.7 V to 0.9 V (OER region) are both important for the growth of the Ir hydrous oxide. To be specific, anodic sweeps to 0.9 V and cathodic to −0.8 V vs. SCE must be embraced by the potential window, so that the in situ fabrication method of an Ir wire responds well to pH.

### 3.3. pH Dependency of the Voltammetric Responses of the Iridium Hydrous Oxide Layers

Following the activation of the Ir wire by potential cycling under the potential window optimised above, the pH dependencies of the two redox signals within the iridium hydrous oxide layer (Ir^3+/4+^, ${\mathrm{IrO}}_{x}O^{I-}/{\mathrm{IrO}}_{x}O^{\mathrm{II}-}H$) and the H underpotential deposition are investigated for the pH range 7.5–8.5 in this section. Linear sweep voltammetry (LSV) was first applied, followed by square wave voltammetry (SWV) to explore the relative sensitivity of the pH dependency to the two techniques.

#### 3.3.1. Linear Sweep Voltammetry

**Figure 4:**
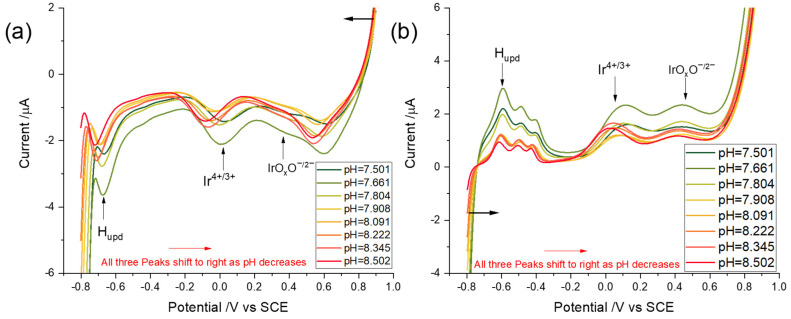
Linear sweep voltammograms with a scan rate of 0.5 Vs^−1^ using an activated iridium wire electrode with varying pH of different buffers ranging from 7.50 to 8.50 (**a**) reduction and (**b**) oxidation.

Prior to the measurements, the pHs of buffers with pH = 7.50–8.50 were measured by a pH meter calibrated by ‘standard seawater buffers’, which were defined as discussed above, using the total hydrogen ion scale with an uncertainty of 0.01. LSVs were first scanned cathodically from 0.90 to −0.80 V vs. SCE at a scan rate of 0.5 Vs^−1^ obtaining reduction peaks for ${\mathrm{IrO}}_{x}O^{I-}$ and Ir^4+^ and for the formation of adsorbed (upd) hydrogen (Figure 4a). Then, scans were immediately reversed to 0.90 V generating the corresponding oxidation peaks (Figure 4b). All measurements were repeated more than three times. The pH-sensitive redox couples obtained in synthetic seawater in the range of pH from 7.50–8.50 had peak potentials which shifted towards more negative potentials as the pH increased for both oxidative and reductive scans. The reduction peak of Ir^4+^ shifted from ca 0.018 to −0.084 V when pH increased from 7.50 to 8.50, while H_upd_ signal moved from −0.66 to −0.72 V (Figure 4a). For re-oxidation peaks, that of Ir (III) occurred at ca 0.097 V vs. SCE for pH = 7.50, and then shifted to 0.011 V when pH = 8.50, while the signals of H desorption moved from −0.59 V to −0.61 V when pH increased from 7.50 to 8.50. The ${\mathrm{IrO}}_{x}O^{I-}/{\mathrm{IrO}}_{x}O^{\mathrm{II}-}H$ LSV redox peak obtained by CV was not apparent by LSV so that its pH dependency was not studied in this section. Thus, SWV was used to increase the signal sensitivity, which will be discussed in the next section.

**Figure 5:**
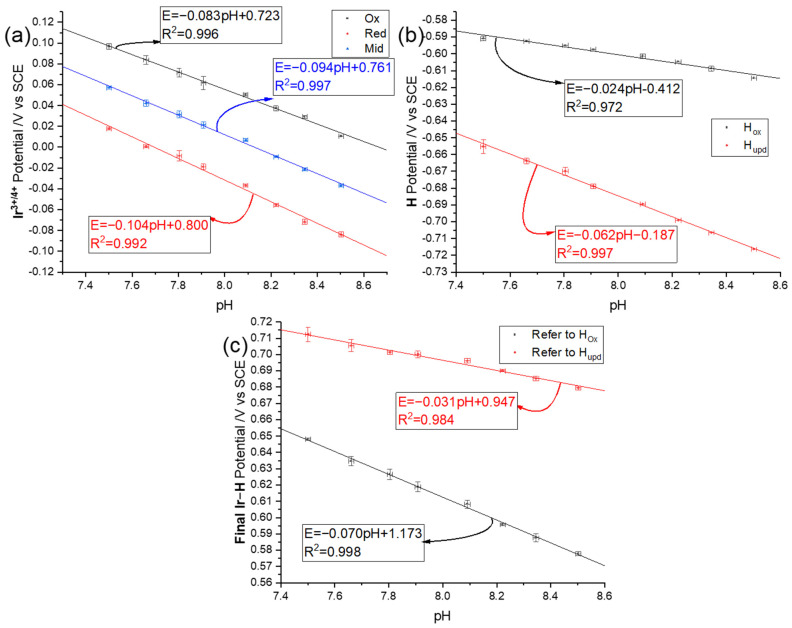
Plots of oxidation (black dots), reduction (red dots) peak, and midpoint (blue dots) potentials against pH value with the latter read from a pH meter (defined using total hydrogen ion scale) (**a**) Ir^III/IV^ (**b**) H_upd/ox_, and (**c**) Ir^III/IV^ midpoints pH dependency referring to either H_upd_ or H_ox_; x-axis error-bar is the uncertainty of pH measured by a pH meter.

To analyse the data, the oxidation and reduction peak potentials of Ir(III/IV) (Figure 5a) and H_upd_/H_ox_ were recorded with the corresponding midpoint potentials of Ir(III/IV) being calculated. Ir(III/IV) redox reaction showed a super-Nernstian relationship (93.7 ± 2.1 mV per pH unit), which is a clear merit of the iridium oxide approach to pH sensing. Meanwhile, the super-Nernstian pH dependency agrees with the equilibria proposed by Olthuis et al. [65] as indicated in Equation (10), namely three proton–two electron transfer reaction. In Figure 5b, the H_upd_ peak showed a near-Nernstian response (62.3 ± 1.5 mV per pH unit). As discussed in Section 1, the pH dependency is consistent with the expected one proton–one electron transfer [74,75]. The best-defined reoxidation peak pointed by an arrow was studied and resulted in less pH sensitivity (23.6 ± 1.6 mV per pH unit). To develop a calibration-free sensor, the super-Nernstian redox couple of Ir(III/IV) was reported relative to one or another of the less pH-sensitive H redox signals (Equation (14) or Equation (15)):

(14) $$y_{\mathrm{potential}}={\mathrm{mid}}_{\mathrm{Ir}}-E\left(H_{\mathrm{ox}}\right)$$

(15) $$y_{\mathrm{potential}}={\mathrm{mid}}_{\mathrm{Ir}}-E\left(H_{\mathrm{upd}}\right)$$

In this way, the reported response becomes independent of the reference electrode value and hence of any drift in the latter, for example, because of electrode fouling or variable liquid junction potentials. Figure 5c shows the experimental data analysed according to both Equations (14) and (15). The slope using H_ox_ as the reference signal was 70.1 ± 1.4 mV per pH unit, while it was 31.3 ± 1.6 mV per pH unit referring to H_upd_ peak potentials. One can conclude that referring to H_ox_ results in a higher pH sensitivity with a smaller uncertainty. In the next section, SWV was explored to identify any possible improvements in the analytical responses.

#### 3.3.2. Square Wave Voltammetry

**Figure 6:**
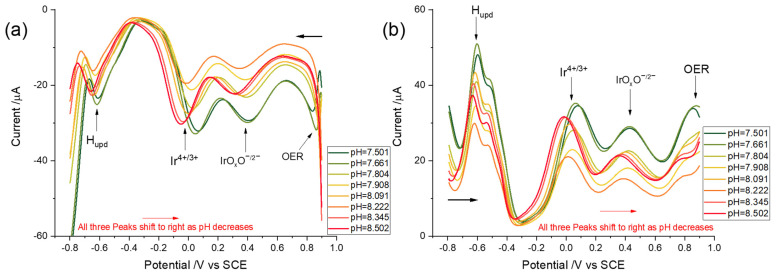
Square wave voltammograms (frequency 90 Hz, step potential 1 mV, and amplitude 60 mV) response using an activated iridium wire electrode in synthetic seawater with varying pHs ranging from 7.50 to 8.50 (**a**) reduction and (**b**) oxidation.

In this section, the signal-to-background ratio of the redox peaks of interest was explored using square wave voltammetry (SWV) following potential cycling activation of the iridium wire. The optimisation of the SWV parameters, including frequency, step potential, and amplitude, was implemented to obtain the best-defined square wave voltammograms for pH measurements as identified elsewhere [48]. The optimised SWV parameters, namely a frequency of 90 Hz, an amplitude of 60 mV, and a step potential of 1 mV, were applied for pH measurements in synthetic seawater with various pHs. First, an iridium wire was activated by potential cycling activation with a potential window between −0.80 V and 0.90 V vs. SCE at a scan rate of 0.5 Vs^−1^ for 40 cycles in synthetic seawater solutions (pH = 7.50–8.50). Then, SWVs with optimised parameters were conducted following in situ activation. All measurements were repeated three times. The reduction peaks were recorded first, which initially swept to the negative potential, −0.80 V vs. SCE, from 0.90 V (Figure 6a), and the scans were then reversed to obtain oxidation peaks (Figure 6b). The peak potentials of the resulting pH-sensitive redox couples shifted towards more negative potentials as the pH increased for both oxidative and reductive scans. Interestingly, whilst a peak attributable to the ${\mathrm{IrO}}_{x}O^{I-}/{\mathrm{IrO}}_{x}O^{\mathrm{II}-}H$ redox couple was not apparent in the linear sweep voltammetry, it was apparent in the SWV because of the increased resolution. The redox couples occurred at similar potentials as observed in the CVs, with physically insignificant differences of the order of 10^−3^ V. To be more specific in terms of pH dependency, the reduction peak occurred at ca 0.42 V vs. SCE in a pH = 7.50 seawater and shifted to ca 0.33 V when the pH was 8.50. Meanwhile, the reduction peak of Ir^4+^ shifted from ca 0.044 to −0.062 V, and the H_upd_ signal moved from −0.60 to −0.65 V when the pH increased from 7.50 to 8.50 (Figure 6a). In the cases of the reoxidation peaks, that of ${\mathrm{IrO}}_{x}O^{\mathrm{II}-}H$ appeared at ca 0.44 V vs. SCE for pH = 7.50 and shifted to 0.36 V when pH = 8.50, while the Ir(III) oxidation peaks were shifted from ca 0.058 to −0.027 V. The signals of H oxidation moved from −0.59 V to −0.63 V when pH increased from 7.50 to 8.50 (Figure 6b). Note that capacitive effects can cause illusory peak-like features in addition to OER at high potentials by SWV, which do not appear on LSV.

**Figure 7:**
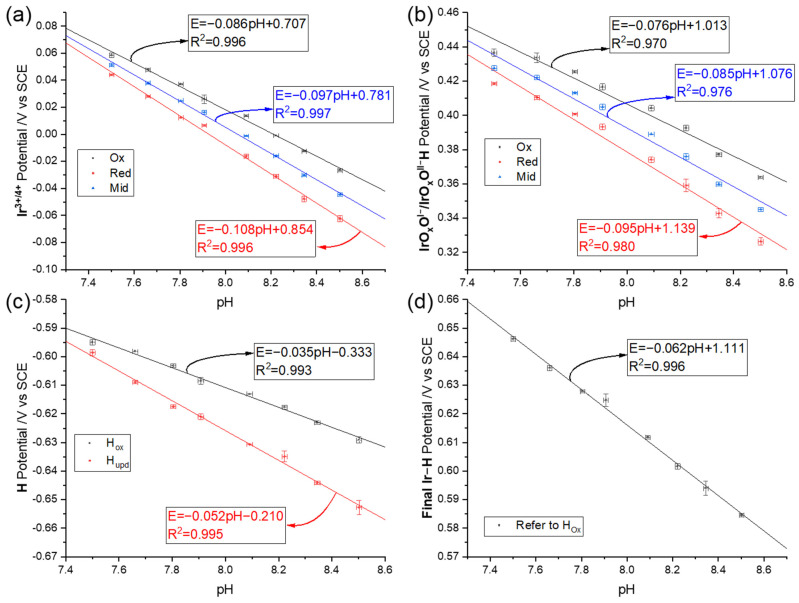
Plots of oxidation (black dots), reduction (red dots) peak, and midpoint (blue dots) potentials against pH value with the latter read from a pH meter (defined using total hydrogen ion scale) (**a**) Ir^III/IV^ (**b**)${\;\mathrm{IrO}}_{x}O^{I-}/{\mathrm{IrO}}_{x}O^{\mathrm{II}-}H$ (**c**) H_upd_/H_ox_ and (**d**) Ir^III/IV^ midpoints pH dependency referring to H_ox_; x-axis error-bar is the uncertainty of pH measured by a pH meter.

Figure 7a and ${\mathrm{IrO}}_{x}O^{I-}/{\mathrm{IrO}}_{x}O^{\mathrm{II}-}H$ (Figure 7b) being calculated. The pH dependencies of H_upd_ and H oxidation are shown in Figure 7c. Similar to the results obtained by LSV, Ir(III/IV) showed a super-Nernstian relationship, 96.9 ± 2.0 mV. It is noteworthy that ${\mathrm{IrO}}_{x}O^{I-}/{\mathrm{IrO}}_{x}O^{\mathrm{II}-}H$ redox reactions also showed a super-Nernstian pH dependency (85.4 ± 5.4 mV per pH unit), which is in contrast to the one proton–one electron transfer mechanism (Nernstian) proposed by Pfizer (Equation (11). However, because the formed iridium hydrous oxide is amorphous, it is difficult to deduce the precise mechanism and stoichiometric composition [81,83,84]. For consistency and convenience, we refer to this redox couple as ${\mathrm{IrO}}_{x}O^{I-}/{\mathrm{IrO}}_{x}O^{\mathrm{II}-}H$ in the following but note the uncertainty in assignment. The H_upd_ peak showed a close Nernstian relationship (52.0 ± 1.6 mV per pH unit), again consistent with a one proton–one electron transfer mechanism [74,75], while the best-defined reoxidation peak highlighted by the arrow in Figure 7 had a weaker pH sensitivity, being 34.7 ± 1.2 mV per pH unit. Equation (14) was again used to calculate the pH dependency to facilitate calibration-free sensing using SWV, being 61.8 ± 1.7 mV per pH unit referring to H_ox_. It was concluded that SWV offered no benefit over LSV, with a small loss of sensitivity and a slightly higher uncertainty and requiring more complex and costly instrumentation.

## 4. Conclusions

We have made and validated a bespoke pH sensor for use in seawater based on an iridium wire electrode. The pH sensitive Ir oxide electrode is formed and activated using an in situ fabrication method in synthetic seawater under neutral conditions by potential cycling. This method can facilitate, by virtue of the in situ method of formation, remote and more diverse pH measurements in contrast to formation via electrodeposition [47,85,86], sol-gel [57,58] chemistry, sputtering [59,60], or thermal methods [61,62], which often require complex conditions and processes; notably, a pre-treatment, hydroxylation [47,87,88], is required for the latter three methods. The optimised potential cycling regime creates three pH-sensitive redox couples on the electrode surface, namely Ir(III/IV), H_upd_/H_ox_, and ${\mathrm{IrO}}_{x}O^{I-}/{\mathrm{IrO}}_{x}O^{\mathrm{II}-}H$. The former two are used so as to realise a calibration-free measurement as coded in Equation (14). The combination of the measurements removes the effects of drift of the reference electrode since both couple as measured almost simultaneously relative to the same arbitrary reference electrode and the difference of the potentials leads to a calibration-free pH sensor, responding to the total pH scale [18,30], for use in seawater showing a super-Nernstian response of 70.1 ± 1.4 mV per pH unit at 25 °C over the pH range of 7.50 to 8.50 corresponding to the usual range found in seawater [8,43].

## Figures and Tables

**Table 1:** Chemical composition of synthetic seawater.

	Constituent	Moles	Weight/g in 0.5 L	Final pH
Synthetic Seawater	NaCl	0.388	11.32	N/A
	KCl	0.011	0.39	
	MgCl_2_	0.055	2.61	
	CaCl_2_	0.011	0.60	
	Na_2_SO_4_	0.029	2.08	
	HCl	0.04	1.73 (mL in vol.)	
One of	2-Aminopyrine	0.08	3.76	6.77
	Tris	0.08	4.84	8.07
	Bis	0.08	4.20	8.81

## Data Availability

The study did not report any data.

## Supplementary Materials

The following supporting information can be downloaded at: https://www.mdpi.com/article/10.3390/s22093286/s1, Table S1: Performance of iridium oxide-based pH electrodes made by different methods. References [89,90] are cited in the Supplementary Materials.
