# Supplementary material for: A Calibration-Free pH Sensor Using an In-Situ Modified Ir Electrode for Bespoke Application in Seawater
Source: Sensors (Basel). 2022 Apr 25;22(9):3286. doi: 10.3390/s22093286 (PMC9102169; doi:10.3390/s22093286)
Supplement: Supplementary file 1 [file sensors-22-03286-s001.zip › sensors-1652349-supplementary.pdf]

sensors MDPI

Supporting Information

Article

A Calibration-Free pH Sensor Using an In-Situ Modified Ir Electrode for Bespoke Application in Seawater

Yuqi Chen and Richard Compton *

Physical & Theoretical Chemistry Laboratory, University of Oxford, Oxford OX1 3QZ, UK; yuqi.chen@sjc.ox.ac.uk
* Correspondence: richard.compton@chem.ox.ac.uk; Tel.: +44-(0)-1865-275957; Fax: +44-(0)-1865-275-410-1

Section S1 Performance of iridium oxide-based pH electrodes made by different methods.

Table S1. *Performance of iridium oxide-based pH electrodes made by different methods.*

Fabrication method	pH dependency	Authors/Year
Electrodeposition	101 mV per pH unit	Chaisiwamongkhol et al.[1]/2019
Electrodeposition	63-82 mV per pH unit	Baur et al.[2]/1998
Sol-gel	48-59 mV per pH unit	Nguyen et al.[3]/2015
Sol-gel	51 mV per pH unit	Huang et al.[4]/2011
Sputtering	59 mV per pH unit	Kuo et al.[5]/2013
Sputtering	54 mV per pH unit	Kreider et al.[6]/1995
Thermal	59 mV per pH unit	Ardizzone et al.[7]/1981
Thermal	59 mV per pH unit	Hitchman et al.[8]/1992
(AIROF)	71 mV per pH unit	Kinoshita, E. et al.[9]/1986
(AIROF)	75 mV per pH unit	Hitchman et al.[10]/1988
(AIROF)	62-74 mV per pH unit	Olthuis et al.[11]/1990

The iridum oxide is one of the most popular candidates for pH sensors, which can be generated by different methods [12, 13]. Electrodeposition is one of the methods and uses a solution of potassium hexachloroiridate(III) as a precursor [1, 2]. Iridium oxides can also be prepared by sol-gel [3, 4] chemistry, sputtering [5, 6], or thermal methods [7, 8]. Cyclic voltammetry is a method of the anodic iridium oxide film (AIROF) synthesis [9-11], which was applied in this paper. Note that the redox mechanism and corresponding pH response of the synthesised Ir oxide electrode is highly dependent on the fabrication method as shown in Table S1.

References

1. Chaisiwamongkhol, K.; Batchelor-Mcauley, C.; Compton, R.G. Optimising amperometric pH sensing in blood samples: an iridium oxide electrode for blood pH sensing. *Analyst* **2019,** 144, 1386–1393.
2. Baur, J.E.; Spaine, T.W. Electrochemical deposition of iridium (IV) oxide from alkaline solutions of iridium (III) oxide. *J. Electroanal. Chem.* **1998,** 443, 208–216.
3. Nguyen, C.; Rao, S.; Yang, X.; Dubey, S.; Mays, J.; Cao, H.; Chiao, J.-C. Sol-Gel Deposition of Iridium Oxide for Biomedical Micro-Devices. *Sensors* **2015,** 15, 4212–4228.
4. Huang, W.-D.; Cao, H.; Deb, S.; Chiao, M.; Chiao, J.C. A flexible pH sensor based on the iridium oxide sensing film. *Sens. Actuators A: Phys.* **2011,** 169, 1–11.
5. Kuo, L.-M.; Chou, Y.-C.; Chen, K.-N.; Lu, C.-C.; Chao, S. A precise pH microsensor using RF-sputtering IrO2 and Ta2O5 films on Pt-electrode. *Sens. Actuators B: Chem.* **2014,** 193, 687–691.
6. Kreider, K.G.; Tarlov, M.J.; Cline, J.P. Sputtered thin-film pH electrodes of platinum, palladium, ruthenium, and iridium oxides. *Sens. Actuators B: Chem.* **1995,** 28, 167–172.
7. Ardizzone, S.; Carugati, A.; Trasatti, S. Properties of thermally prepared iridium dioxide electrodes. *J. Electroanal. Chem. Interfacial Electrochem.* **1981,** 126, 287–292.
8. Hitchman, M.L.; Ramanathan, S. A field-induced poising technique for promoting convergence of standard electrode potential values of thermally oxidized iridium pH sensors. *Talanta* **1992,** 39, 137–144.
9. Kinoshita, E.; Ingman, F.; Edwall, G.; Thulin, S.; Głąb, S. Polycrystalline and monocrystalline antimony, iridium and palladium as electrode material for pH-sensing electrodes. *Talanta* **1986,** 33, 125–134.
10. Hitchman, M.L.; Ramanathan, S. Evaluation of iridium oxide electrodes formed by potential cycling as pH probes. *Analyst* **1988,** 113, 35–39.
11. Olthuis, W.; Robben, M.A.M.; Bergveld, P.; Bos, M.; Van Der Linden, W.E. pH sensor properties of electrochemically grown iridium oxide. *Sens. Actuators B Chem.* **1990,** 2, 247–256.
12. Głab, S.; Hulanicki, A.; Edwall, G.; Ingman, F. Metal-Metal Oxide and Metal Oxide Electrodes as pH Sensors. *Crit. Rev. Anal. Chem.* **1989,** 21, 29–47.
13. Yao, S.; Wang, M.; Madou, M. A pH electrode based on melt-oxidized iridium oxide. *J. Electrochem. Soc.* **2001,** 148, H29.
